# Vibrational Properties of Pd Nanocubes

**DOI:** 10.3390/nano9040609

**Published:** 2019-04-12

**Authors:** Alberto Flor, Juan M. Feliu, Chia-Kuang Tsung, Paolo Scardi

**Affiliations:** 1 University of Trento, Department of Civil, Environmental and Mechanical Engineering, via Mesiano 77, 38123 Trento, Italy; alberto.flor@unitn.it; 2 Institute of Electrochemistry, University of Alicante, Carretera de San Vicente del Raspeig s/n, 03690 Alicante, Spain; juan.feliu@ua.es; 3 Boston College, Merkert Chemistry Center, Department of Chemistry, 2609 Beacon St., Chestnut Hill, 02467 MA, USA; frank.tsung@bc.edu

**Keywords:** Vibrational properties of nanocrystals, metal nanocrystals, nanocubes, mean square displacement, Debye–Waller coefficient, X-ray diffraction, molecular dynamics

## Abstract

The atomic disorder and the vibrational properties of Pd nanocubes have been studied through a combined use of X-ray diffraction and molecular dynamics simulations. The latter show that the trend of the mean square relative displacement as a function of the radius of the coordination shells is characteristic of the nanoparticle shape and can be described by a combined model: A correlated Debye model for the thermal displacement and a parametric expression for the static disorder. This combined model, supplemented by results of line profile analysis of the diffraction patterns collected at different temperatures (100, 200, and 300 K) can explain the observed increase in the Debye–Waller coefficient, and shed light on the effect of the finite domain size and of the atomic disorder on the vibrational properties of metal nanocrystals.

## 1. Introduction

The mean square displacement (MSD, 〈*u*^2^〉) provides a direct connection between experimental observations and atomistic models for the study of the vibrational properties of crystalline phases. In fact, MSD can be obtained from different spectroscopic techniques, including (X-ray and neutron) scattering [1], X-ray absorption spectroscopy (XAS) [2], Raman spectroscopy [3,4], as well as low energy electron diffraction (LEED) [5] and Mössbauer spectroscopy [6], which focus more selectively on the contribution of the surfaces. In the field of atomistic modelling, on the other hand, the MSD is directly obtainable from simulations of molecular dynamics (MD), tracing atomic positions over time [7], to represent the vibrational dynamics of the systems studied.

In monoatomic systems such as metal nanocrystals, the MSD provides a direct estimate of the average energy of the vibration modes, *E*. If the metal atom is approximated as an Einstein oscillator, of independent mass *m* and average vibration frequency ω, the MSD is [8]:

(1) $$\langle u^{2}\rangle =\frac{3E}{m\omega^{2}}=\frac{3\hbar }{m\omega }\left[\frac{1}{2}+n\left(\omega ,T\right)\right]≃\frac{3k_{B}T}{m\omega^{2}},$$

where n(ω,T) is the phonon number, given by the Bose–Einstein distribution. From here it is quite straightforward to identify the link between MSD and thermal capacity, and other thermodynamic quantities characteristic of the nanocrystal. It is of course possible to write expressions more complete than Equation (1), taking into account the whole phonon spectrum, summing over the normal modes of vibration, considering the dispersion relations, the correlation of the displacements of neighboring atoms, as well as anisotropy and anharmonicity of the atomic potentials [1,8]; however, the main result expressed by the last equality in Equation (1), proportionality to *k_B_T*, remains valid in the limit of high temperatures.

Concerning metal nanocrystals, it has long been known that the MSD is greater than in the corresponding large crystals [9,10,11]. MSD increase is generally assumed to go with 1/*D*, where *D* is any characteristic length in the nanocrystal [12], and is attributed to a surface softening, caused by the different arrangement and under-coordination of the atoms of the surface.

A larger MSD means a higher Debye–Waller (DW) coefficient ($B_{ISO}=8\pi^{2}\langle u^{2}\rangle /3\;$), which is often translated into a lower Debye temperature, $\Theta_{D}$. Several experimental observations performed by LEED [9,12,13] and by Mössbauer spectroscopy [6,14] show a lower $\Theta_{D}$ for the surface shell than within the nanoparticle. Based on this evidence core-shell models have been proposed for the interpretation of spectroscopy data (e.g., see [15]), although not always there is direct evidence of a clear cut, a well-defined boundary between core and shell. Indeed, MD simulations of metal nanoparticles provide a more complex picture, with no clear separation between core and shell. Simulations show that properties like the MSD change gradually [16,17], with a gradient from core to surface. Moreover, simulations clearly point out the presence of two components, respectively static and dynamic (thermal), of the MSD [18].

From the experimental point of view, a recent study has shown the difficulty of obtaining reliable values of *B_ISO_* for nanocrystalline powders and agglomerates by means of X-ray diffraction (XRD) [19]. Correct values can only be obtained from data of high statistical quality, collected with radiation energy sufficiently high to limit the effects of absorption and to encompass many diffraction peaks in the reciprocal space. Moreover, it is important to account for the MSD effect both on the Bragg peaks and on the thermal diffuse scattering (TDS); data modelling must also consider the finite size of the diffraction domains, which gives a broadening of the Bragg peaks, but also affects the TDS. In particular, the finite size of the nanocrystals is responsible for a phonon confinement, enforcing an upper bound to the vibration wavelengths, contributing to the characteristic shape of the TDS peaks of nanocrystals [20].

The present work investigates the thermal behavior of Pd nanocubes, about 24 nm edge. The synchrotron radiation beamline 11-BM at the advanced photon source (APS) of Argonne National Laboratory (ANL, Lemont, IL, USA) supports the measurement of high quality XRPD patterns from which *B_ISO_* values can be obtained by modelling the line profiles collected at three temperatures: 100, 200, and 300 K. As expected, DW coefficients are larger than those reported for bulk Pd, and the difference can be explained by varying contributions from the dynamic and the static disorder. MD simulations show the origin of the two contributions, and the role of the correlated displacements of neighboring atoms. The trend of the MSRD (Mean square relative displacement) can be obtained as a function of the radius of the coordination shells in the nanocrystals, showing the effect of the size and shape of the nanocrystals, providing the means to single out static and dynamic contributions. A simple model, requiring just a single adjustable parameter for each contribution, is used to fit the experimental data. While the first model, concerning static disorder, stems directly from the trend of the MSRD obtained from MD simulations, the second one follows a recent extension of the correlated Debye model accounting for the effect of the finite size of the nanocrystals [18].

## 2. Experimental

**Figure 1:**
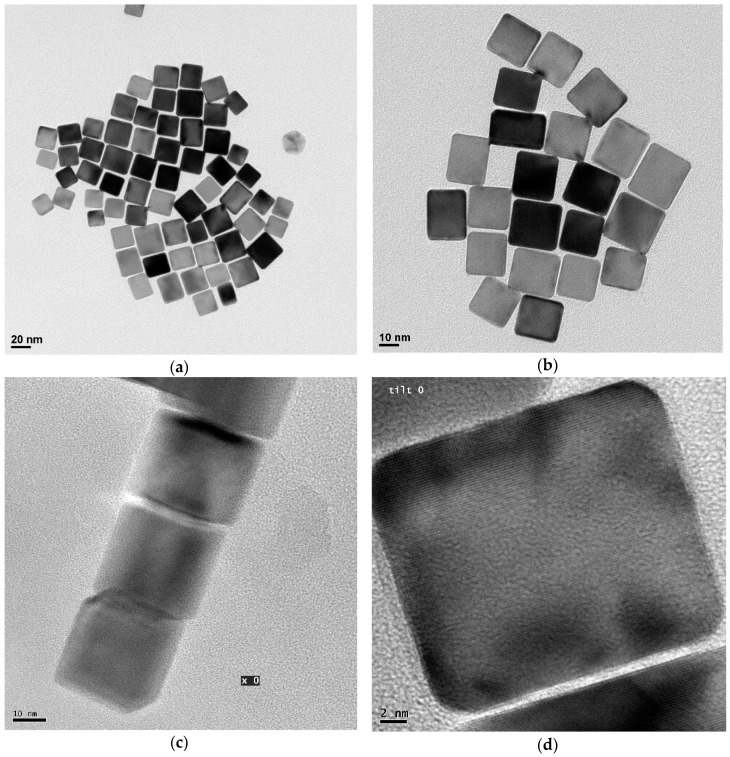
Transmission Electron Microscopy (TEM) images of the Pd nanocubes (see Supporting Information in [23]) From (**a**) to (**d**) progressive magnification to the nanoparticles. Image (**a**) and (**b**) show the limited size dispersion and an example in (**a**) of the non-crystallographic fraction (the multiply-twinned particle on the right of the image). In (**c**) and (**d**) a magnification of a single nanoparticle, showing details of the truncated cube shape. (Images adapted from [23], with permission from *iucr journals*, 2019).

The experimental case study is a powder of Pd nanocrystals prepared according to the procedure indicated by [21,22], based on the reduction of H_2_PdCl_4_ with ascorbic acid in the presence of cetyltrimethylammonium bromide (CTAB, Aldrich 99%). As shown in Figure 1, the nanocrystals are cubes with truncated corners and edges which expose, respectively, {111} and {110} planes in addition to the {100} cube faces. While the shape is rather constant in the powder sample (truncation fraction 0.23, see [23] for full details), transmission electron microscopy (TEM) and XRD have shown that the edge lengths are dispersed according to a lognormal distribution with 23.7 nm mean and 5.3 nm standard deviation (as can be seen in Figure 1a). A minor fraction (~7%) is mainly made of multiply-twinned (so called non-crystallographic) nanoparticles (e.g., see the decahedron in Figure 1a).

Diffraction data were obtained from capillaries (Kapton tubes, 0.8 mm diameter) loaded with concentrated nanocrystal dispersion, allowed to dry for a few days and then sealed with epoxy. Data were collected at 11-BM, the powder diffraction beamline of APS-ANL, using 30 keV radiation (actual wavelength from calibration procedures with standard Si powder: λ = 0.0413692 nm) with the traditional Debye–Scherrer geometry: full details on 11-BM operating conditions can be found in [24] and website of the facility. The capillary, rotated at 4200 rpm, was analyzed at three temperatures: 100, 200, and 300 K; measurements were made on a spot previously selected for the low absorption (I/I_0_ ≤ 0.02), such that no intensity corrections are necessary in the modelling of the patterns.

The XRD patterns collected at the three temperatures were analyzed together, using the whole powder pattern modelling (WPPM) approach in a modified version of the PM2K software (University of Trento, Italy, [25] and references therein). The instrumental profile was acquired by modelling the pattern of standard LaB_6_ powder (NIST SRM-660b), whereas the pattern of the sample holder was obtained from an empty kapton capillary, modelled by pseudoVoigt curves [26] and adapted to the Pd-filled capillary by means of an adjustable scale parameter. 

Size and strain broadening models were the same for the three temperatures: Domain shape, as suggested by TEM, is a truncated cube (thus exposing 110 and 111 facet in addition to the 100 cube faces); the modelling allows the refinement of the truncation level, in addition to the mean and variance of a lognormal distribution of edge lengths [27]; for the strain component the Popa–Adler–Houska (PAH) model has been used [19,25], which adapts well to the case of nanocrystals [28], providing also for the anisotropy of atomic displacement in the nanocubes. Besides scale parameters and coefficients of the (Chebyshev) polynomial background, Pd unit cell parameter and DW coefficients were refined independently, for each of the three patterns, to account for the effect of temperature. *B_ISO_* enters the traditional DW factor decreasing the Bragg scattering intensity, as well as the thermal diffuse scattering (TDS). The TDS was modelled according to [20], to take into account the effect of finite size of the crystalline domains. 

## 3. Atomistic Modelling

### 3.1. Molecular Dynamics, Mean Square Displacement and Mean Square Relative Displacement

Nanocrystal models were constructed by replicating the primitive unit cell (with the unit cell parameter of bulk Pd, *a*_0_ = 0.3890 nm [29]) in ideal atomic arrangements, until the space was filled. A condition was enforced to the atomic coordinates, in order to keep only those atoms belonging to the desired geometrical shape. This system was the starting step for MD simulations. Calculations have been performed with the open source software LAMMPS [30], using the in embedded-atom method (EAM) [31,32] interatomic potential. After the minimization process and the thermalization of the system, the nanoparticle was left to freely evolve at room temperature as a microcanonical statistical ensemble. The atomic coordinates were saved during the last part, for a duration of half a nanosecond. 

MD provides the trajectory in time of each atom in the nanoparticle, $r_{i}\left(t\right)$, from which time average position, $\bar{r_{i}}$, and variance (MSD), $\bar{\sigma_{i}^{2}}$, can be easily derived. Likewise, the distance between any two atoms, $r_{ij}\left(t\right)$, can be used to obtain the MSRD, $\bar{\sigma_{ij}^{2}}$. For monoatomic systems for which $\bar{\sigma_{i}^{2}}$ can be assumed constant throughout the particle (which is clearly an approximation), the two squared displacements are related by:

(2) $$\bar{\sigma_{ij}^{2}}=2\bar{\sigma_{i}^{2}}-2\bar{\sigma_{i}\cdot \sigma_{j}},$$

where the second term is the displacement correlation function (DCF). The DCF accounts for the correlation of atomic motions and is significantly different from zero for the first (innermost) coordination shells. As it is known, the DW coefficient used in traditional XRD models is proportional to the MSD, whereas other spectroscopies, like XAS, provide the MSRD, and a richer information on the local atomic environment of the innermost coordination shells [2,33].

The actual quantities to compare with experimental results must involve a spatial (configuration) average (i.e., an average over all atoms or couples of atoms). From the MD trajectory, the MSRD can be calculated as a function of the number of unique atom pairs, $N_{S_{r}}$, and radius, *r*, of the coordination shells *S_r_* in the nanocrystal

(3) $$\bar{\sigma_{r}^{2}}=\frac{1}{N_{S_{r}}}\sum_{i,j\in S_{r}}\bar{\sigma_{ij}^{2}}=\langle \bar{\sigma_{ij}^{2}}\rangle_{r}.$$

MD also provides the means to single out the static component of displacement, in a way inaccessible to experiments. In fact, given the MD trajectory, made of a series of “snapshots” of the nanoparticle in time, the spatial average of Equation (3) can be calculated for the time average of distances, $\bar{r_{ij}}$:

(4) $$\sigma_{0,r}^{2}=\frac{1}{N_{S_{r}}}\sum_{i,j\in S_{R}}{\left(\bar{r_{ij}}-\frac{1}{N_{S_{r}}}\sum_{i,j\in S_{R}}\bar{r_{ij}}\right)}^{2}={\langle {\left(\bar{r_{ij}}-\langle \bar{r_{ij}}\rangle \right)}^{2}\rangle }_{r}.$$

While Equation (3) provides the overall MSRD, including all (static and dynamic) components of the displacement, Equation (4) gives the static component only. It is; therefore, possible to obtain a good estimate of the dynamic (thermal) component alone from the difference between Equations (3) and (4), assuming that the two MRSDs can be treated as variances of independent distributions:

(5) $$\bar{\sigma_{T,r}^{2}}≃\bar{\sigma_{r}^{2}}-\sigma_{0,r}^{2}=\langle \bar{r_{ij}^{2}}\rangle_{r}-2\langle {\bar{r_{ij}}}^{2}\rangle_{r}+\langle \bar{r_{ij}}\rangle_{r}^{2}.$$

**Figure 2:**
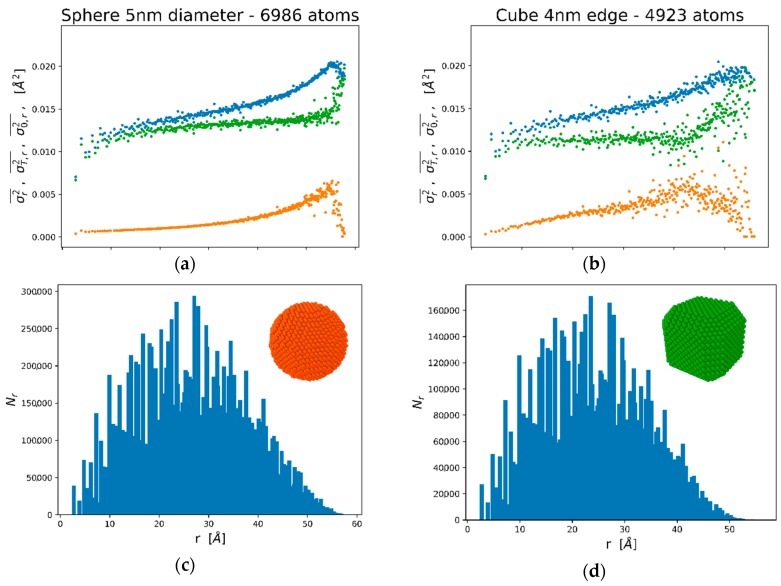
Trends of the Mean Square Relative Displacement (MSRD) for a spherical (**a**) and truncated cube (**b**) crystal, N = 6986 and N = 4923 Pd atoms, respectively; corresponding number of atom pairs (*N_r_*) is shown by histograms in (**c**) and (**d**). MSRD is shown for the total (blue), static (orange), and thermal (green) components, as of Equation (3), (4), and (5), respectively. Insets in (**c**) and (**d**) show images of the nanoparticle shapes.

**Figure 3:**
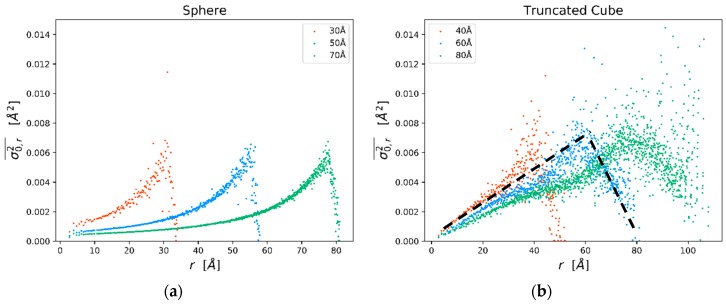
Static MSRD component from Molecular Dynamics (MD) simulations for three spheres (**a**) and three truncated cubes (**b**) of increasing size. The dotted line is the trend according to the parametric model of Equation (7).

Figure 2a shows the trend of the MSRD, according to Equations (3)–(5), as a function of *r* in a spherical Pd nanoparticle of about 5 nm diameter (N = 6986 atoms). Similar calculations can be performed for any desired particle shape. Figure 2c,d refer to a Pd cube, about 4 nm edge (N = 4923 atoms), with truncated corners and edges. This is the shape suggested by TEM images and by the modelling of the XRD patterns of this study; indeed, the extent of the truncation is that obtained by WPPM (see below), while the size in the simulation is smaller than in the experimental sample to limit the computation time. The last is not a limitation because, quite interestingly, the trends are characteristic of the nanoparticle shape: By increasing the nanocube size the MSRD trends are the same, provided that the abscissa (shell radius) is stretched to match the edge length (or the diameter for spherical nanoparticles). The scalability of the MSRD trends is demonstrated in Figure 3 for the static MSRD component (Equation (4)) in a selection of spheres and truncated cubes of increasing size.

As already pointed out, different spectroscopies can access different aspects of the disorder and the vibrational characteristics of the nanocrystals. XRD generally gives the MSD, which can be related to the average of the trends in Figure 2, weighted over $N_{S_{r}}$,

(6) $$B_{ISO}=\frac{8}{3}\pi^{2}\langle u^{2}\rangle ≃8\pi^{2}\frac{1}{N_{S}}\sum_{r}^{r_{MAX}}N_{S_{r}}\frac{\bar{\sigma_{r}^{2}}}{2},$$

where $N_{S}=\sum_{r}^{r_{MAX}}N_{S_{r}}=N\frac{\left(N-1\right)}{2}$ is the total number of unique atom pairs in a crystal with *N* atoms. Equation (6) can also be used to assess the separate contributions of the static or the thermal components, as better illustrated in the following.

### 3.2. Modelling the MSRD Components

While MD simulations provide full details of disorder for each coordination shell, experiments have a more limited scope, and generally allow the measurements of fewer, global parameters, like *B_ISO_* in Equation (6). However, by introducing some reasonable approximations, the MSRD trends can also be related to the experimental evidence from XRD. The thermal component, $\bar{\sigma_{T,r}^{2}}$, can be described by a correlated Debye (CD) model [34,35], recently adapted to nanocrystals, to account for the effect of a small domain size [18] (see also Appendix): given the number of atoms (or equivalently, the nanoparticle size and shape) and temperature, the trend of $\bar{\sigma_{T,r}^{2}}$ can be modelled by adjusting a single parameter, the Debye temperature.

**Figure 4:**
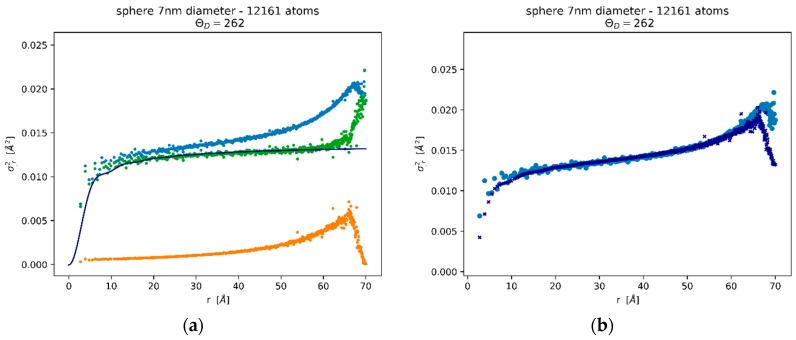
MSRD trends for a spherical Pd nanocrystal with 7 nm diameter (N = 12161 Pd atoms): total (blue), static (orange), and thermal (green) components of MSRD, with the best fit of the Correlated Debye (CD) model (line) (**a**). The sum of CD model and static component (dark blue) is compared with the total MSRD (blue) (**b**).

As an example, Figure 4a shows the data for a spherical Pd nanoparticle of diameter 7 nm, with N = 12161 Pd atoms. Besides the two (static and dynamic) components and the total MSRD, the figure also shows the trend of the CD model. The best fit of the thermal MSRD component was obtained for $\Theta_{D}\;$= 262 K, in agreement with the results of [18] for a system of Pd nanoparticles from 3 to 20 nm. The value is not far from the experimental value of 272 K [36]. The difference is justified, by inevitable discrepancies between EAM potential and reality, but also by the fact that Pd atoms on the surface vibrate with a larger amplitude than in the core, so that the average MSD and MSRD increase slightly, and consequently $\Theta_{D}$ decreases. In Figure 4b we can see that the sum of CD model and static MSRD (dark blue symbol) matches quite well the total MSRD from the MD simulation. Discrepancies are observed for more distant shells, involving atoms on the surface which, as already pointed out, have a larger vibration amplitude. However, given the decaying trend with distance of the weight function $N_{S_{r}}$ (Figure 2b,d), the effect of such deviations on observables like *B_ISO_* (Equation (6)) is small.

For the static component we can exploit the scalability property demonstrated in Figure 3: $\bar{\sigma_{0,r}^{2}}$ values start from zero at *r* = 0, go through a maximum and then drop to zero again for the longest distance in the particle. As already pointed out and shown in Figure 3, changing the size has only the effect of stretching the trend to longer distances, whereas the maximum $\bar{\sigma_{0,r}^{2}}$ value is comparable among different sizes.

It is; therefore, tempting to approximate the observed trends for the truncated cubes (Figure 3b) by a simple linear model. The maximum value is found for a distance of the order of the cube edge, i.e., when most of the pairs of atoms are composed of a surface atom and a second distant atom, but in the core region. Static MSRD falls to zero for the maximum distance in the nanocube (i.e., when pairs are made of atoms on opposite surfaces, thus displaced in similar way).

In a practical implementation of this model, if the nanocrystal size and shape are known (from simulations or from experiments), the trends in Figure 3 (see the dotted line) can be easily parameterized leaving the maximum, $\sigma_{0,max}^{2}$, as the only adjustable parameter:

(7) $$\bar{\sigma_{0,r}^{2}}=\{\begin{array}{cc} \sigma_{0,max}^{2}\left(r/D\right) & if\;0\leq r<D\\ \sigma_{0,max}^{2}\left(r-D_{max}\right)/\left(D-D_{max}\right) & if\;D\leq r<D_{max}\\ 0 & if\;r\geq D_{max} \end{array}.$$

Cube edge (*D*) and maximum distance in the truncated cube (*D_max_*) are obtained from the size broadening of the Bragg peaks in the experimental XRD patterns. Indeed, WPPM refines values of mean size (*D*) and truncation level, from which total number of atoms and *D_max_* are readily determined. Examples of fit in practical cases are shown in detail in the Appendix.

In this way, the experimental values of *B_ISO_* obtained in the present study at different temperatures can be matched by a model with the maximum static MSRD, $\sigma_{0,max}^{2}$, and the Debye temperature for the thermal MSRD, as adjustable parameters:

(8) $$B_{ISO}^{\exp t}\left(T\right)≃4\pi^{2}\frac{1}{N_{S}}\sum_{r}^{r_{MAX}}N_{S_{r}}\left[\bar{\sigma_{T,r}^{2}}\left(T;N,\Theta_{D}\right)+\bar{\sigma_{0,r}^{2}}\left(N,\sigma_{0,max}^{2}\right)\right].$$

## 4. Results and Discussion

**Figure 5:**
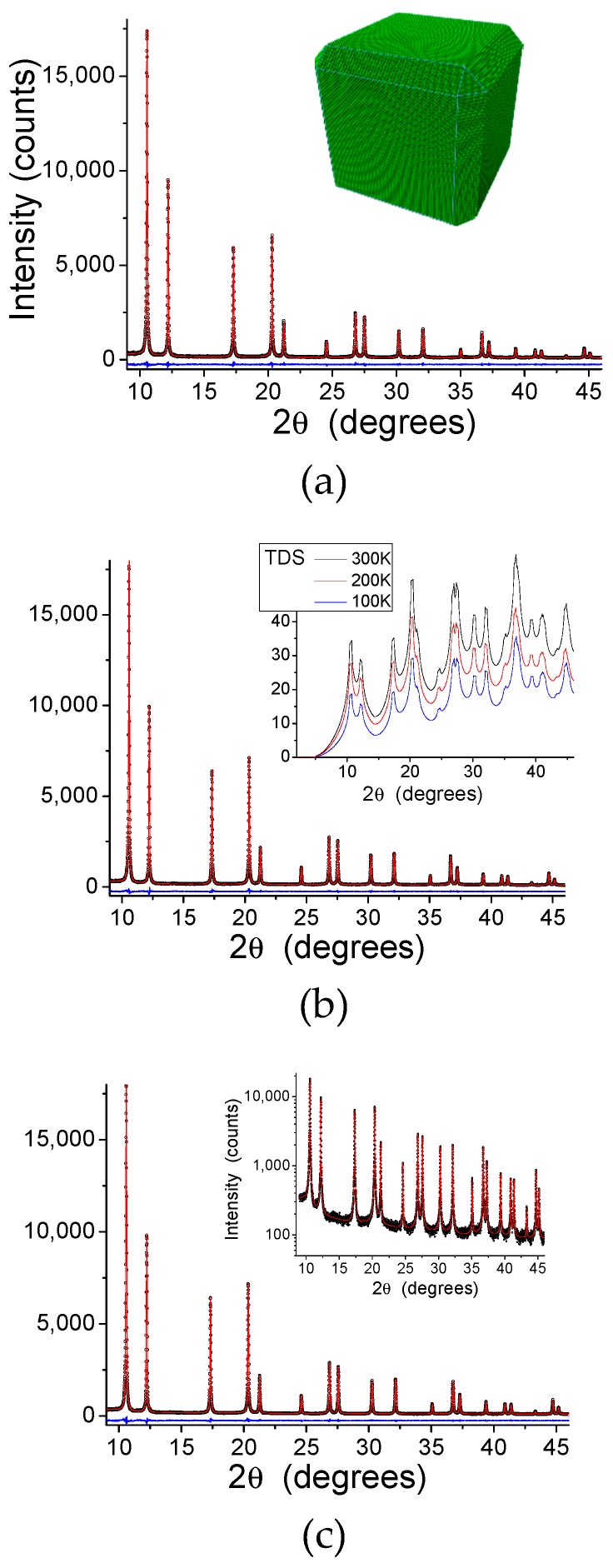
Whole powder pattern model (WPPM) results. X-Ray Diffraction (XRD) patterns (circle), model (line), and difference (residual, line below) at 300 (**a**), 200 (**b**), and 100 K (**c**). Insets: in (**a**), refined shape of the truncated nanocube; in (**b**), Thermal Diffuse Scattering (TDS) component at 100 (blue), 200 (red), and 300 K (black); in (**c**), intensity log scale plot.

**Figure 6:**
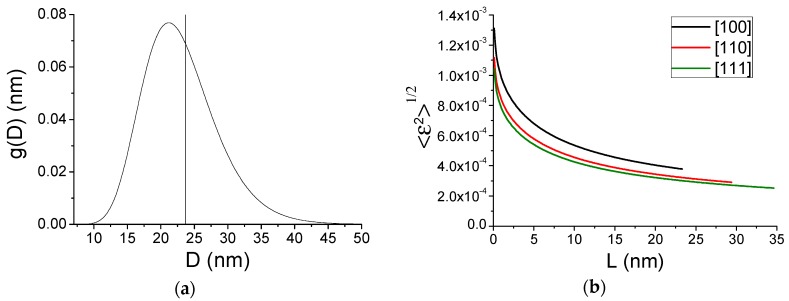
WPPM results. Lognormal distribution of cube edges, *D* (**a**) and microstrain distribution along three crystallographic directions: [100] (black), [110] (red), [111] (green) (**b**).

WPPM results for the patterns collected at 100, 200, and 300 K are shown in Figure 5. The inset in (a) shows the refined particle shape, whereas the contribution of the TDS, increasing as expected with the temperature, is shown in (b); the inset in (c) shows details of the peak tail region in intensity log scale. Size and strain broadening (Figure 6) give the lognormal distribution of nanocube edges, *D*, (a) and the microstrain due to the inhomogeneous atomic displacement caused by the surface effect (b). In the microstrain plot of Figure 6b, it is apparent the effect of the elastic anisotropy of Pd: like most *fcc* metals, Pd is stiffer along [hhh] than [h00], so that microstrain is correspondingly higher along [h00] than [hhh], with [hh0] on intermediate level. These results, obtained from the modelling of patterns at three temperatures, are not far from those of [23] obtained from room temperature (RT) data only and with a different X-ray energy.

**Figure 7:**
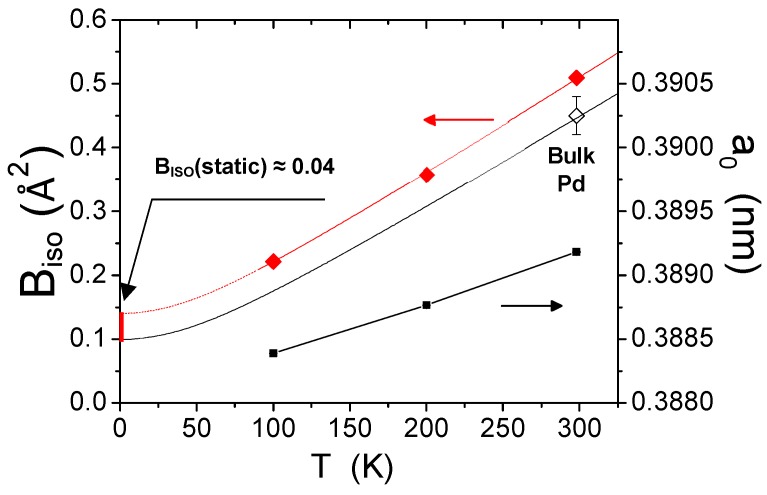
Left axis: Debye-Waller coefficient, *B_ISO_*, from the XRD patterns collected at 100, 200, and 300 K (♦) for the Pd nanocubes and corresponding room temperature (RT) value for bulk Pd (◊) [36]. Red line is the combined model of Equation (8), black line is the extrapolation according to the Debye model for the literature bulk value. The arrow highlights the increase in the static component. Right axis: Unit cell parameter measured from the XRD patterns (▪). See text for details.

Figure 7 shows the results for the temperature dependent parameters, DW coefficient and unit cell parameter. They both increase with the temperature, but while the trend of the unit cell parameter is predictable on the basis of simple thermal expansion, the *B_ISO_* values are significantly higher than those of bulk Pd. This feature, common to many nanocrystalline metals [15,37,38], can be explained both qualitatively and quantitatively with the models discussed above, even though the latter is purely indicative with just three data points; collecting more data on a wider temperature range is priority for our future work.

However, it is interesting to show how the approach works. According to the CD model, the MSRD increases for smaller nanocrystals, for the effect of the decreasing number of atoms and of a reduced Debye temperature for the larger amplitude of the surface atom vibrations [18]; the static component of the MSRD also increases because, as shown in Figure 3, the maximum region weights more on the average (measured) *B_ISO_* for decreasing size. Therefore, both static and dynamic components of the MSRD (and *B_ISO_*) increase for decreasing domain size.

The best fit of the model of Equation (8) is shown as a red line in Figure 7, together with the experimental *B_ISO_* values. As already mentioned, the number of atoms (*N* in Equation (8)) is given by the WPPM, from the mean value of the edge length distribution (Figure 6a), whereas $\sigma_{0,max}^{2}\;$= 0.0015 Å^2^ and $\Theta_{D}$ = 271 K are obtained by the best fit of Equation (8), using the CD model and Equation (7). Once again, we underline that the results are purely indicative, as a credible fit would require more data points over a wider range of temperatures. However, it is quite evident that the refined value of $\sigma_{0,max}^{2}$ is lower than the maximum static MSRD given by the MD simulations (Figure 2, Figure 3 and Figure 4).

The present results suggest that EAM potentials may not be entirely appropriate to describe details of the surface properties of the crystals, which is not a surprise, and indeed a known limitation of EAM [39]. EAM results differ even by changing the potential (e.g., using Sheng potential [40] instead of Zhou potential [41]). But it is also likely that the condition of the surface of real crystals, with the capping CTAB layer and the environment quite different from the vacuum assumed in the MD simulations, also play an important part. More investigations and extensions of the atomistic modelling to account for surface effects will be required, but the general principle can be put forward that DW coefficients of nanocrystals and their vibrational properties should be evaluated both based on dynamic (thermal) and static contributions.

## 5. Conclusions

This work presents an approach to study the vibrational properties of large assemblies of nanocrystals, based on the combined use of a MSRD model and information from the XRD patterns collected at different temperatures. The XRD data, through the whole powder pattern modelling, provide detailed indications on the crystalline domain size and shape, as well as the trend with temperature of unit cell parameter and DW coefficient.

By separating the MSRD of the nanocubes into two contributions, we can highlight the effects of the static component and; therefore, gain a better understanding of the purely thermal proprieties of the nanocrystals. In fact, if the increase in MSRD is entirely attributed to the vibrational part, the Debye temperature tends to be underestimated. The present procedure, instead, returns values for *Θ_D_* that are closer to the bulk value, since part of the deviation in the finite size case is attributed to the static component (i.e., the parameter $\sigma_{0,max}^{2}$ in the parametric model of Equation (7)). Interestingly, the static component of the MSRD is peculiar to the nanoparticle shape (in this work, sphere or truncated cube), thus giving a different perspective on the increase of *B_ISO_*.

Even if not correct in the finer details, like anisotropy and anharmonicity, the proposed model is sufficiently simple and informative to be flexibly used for most nanocrystalline systems, to grasp the main effects of the static and dynamic disorder on spectroscopic results. For best results, *B_ISO_* data should be collected for different values and over a wide temperature range, a condition that requires more experimental efforts in the future.

## Figures and Tables

**Figure A1:**
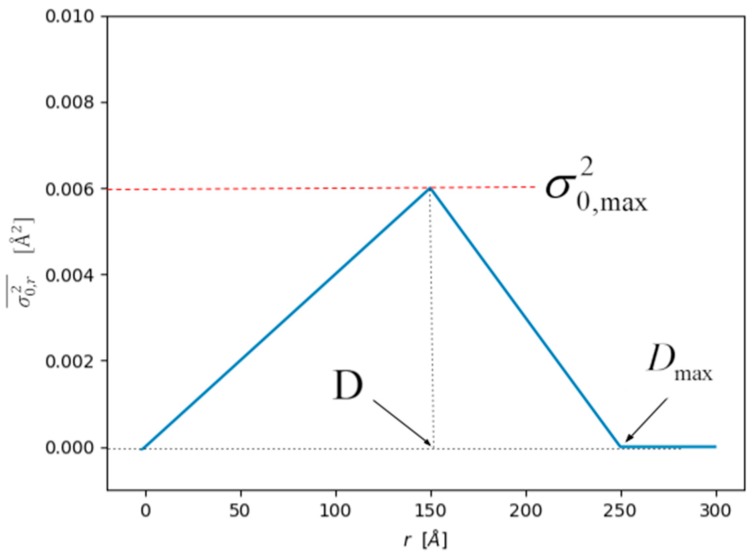
Graphical explanation of Equation (A9). The function is a simplification of the static component trend calculated from MD simulations.

**Figure A2:**
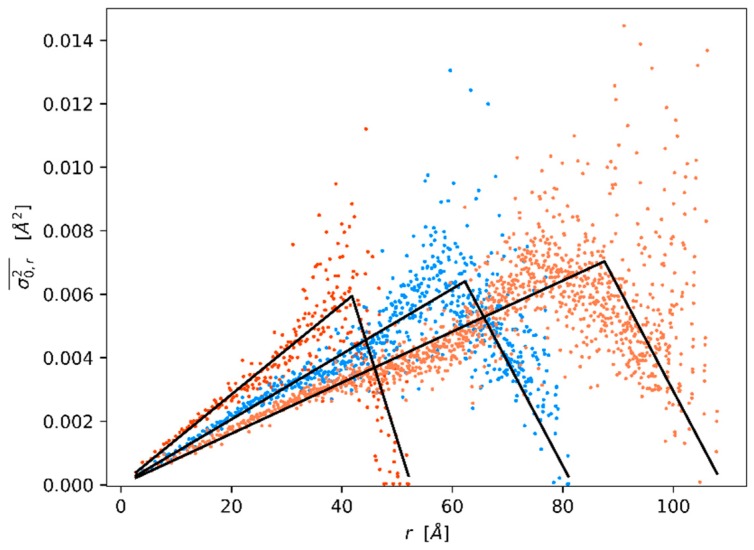
Static MSRD results from Figure 3 in the main text with static model of Equation (A9). Best fit of $\sigma_{0,max}^{2}$ are, respectively, 0.00594 (D = 41.84 Å), 0.00640 (D = 62.26 Å), and 0.00703 (D = 87.54 Å).

**Figure A3:**
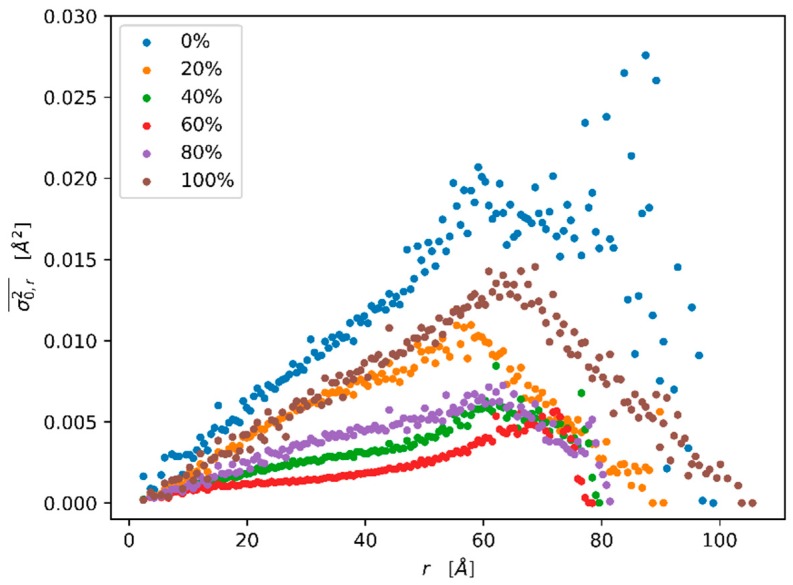
Static MSRD results for nanoparticle of roughly the same number of atoms of a cube of edge 6 nm (15,000 atoms), but with different degrees of truncation (0% is a cube, 100% an octahedron). The overall shape is always similar to the expression A9, except for the case of more “spherical” shapes (truncation 40% and 60%) that exhibit a trend closer to the sphere (Figure 3 in main text). Compared to Figure A3, the data have been presented with larger bins for a better visualization of the trends.

**Figure A4:**
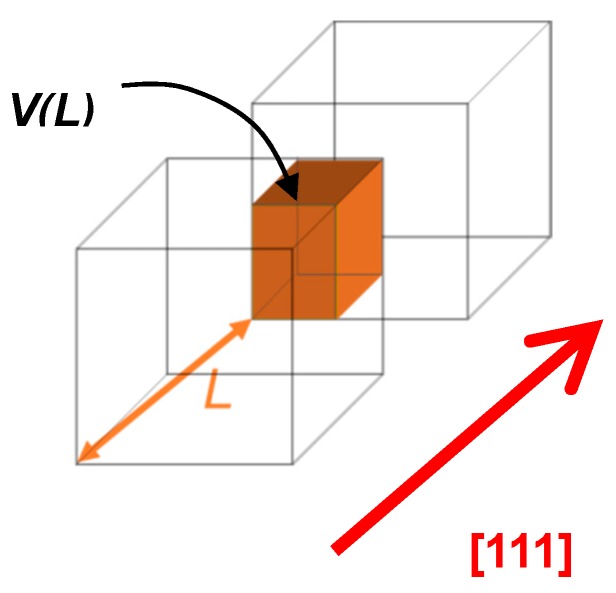
Graphical depiction of the common volume function along direction [111] for a cube.

## Appendix A

### Modified Correlated Debye Model

The modified CD model [18] gives the thermal component of the MSRD of a nanoparticle:

(A1) $$\begin{array}{ll} \bar{\sigma_{T}^{2}}= & A_{1}\{\frac{1}{4}\left[1-{\left(\frac{\Theta_{s}}{\Theta_{D}}\right)}^{2}\right]+{\left(\frac{T}{\Theta_{L}}\right)}^{2}\int_{\frac{\Theta_{s}}{T}}^{\frac{\Theta_{L}}{T}}\frac{xdx}{e^{x}-1}+\frac{\cos\left(\frac{rk_{B}\Theta_{L}}{\hbar c_{m}}\right)-\cos\left(\frac{rk_{B}\Theta_{s}}{\hbar c_{m}}\right)}{2{\left(\frac{rk_{B}\Theta_{L}}{\hbar c_{m}}\right)}^{2}}\\ & -{\left(\frac{T}{\Theta_{L}}\right)}^{2}\Psi_{1}\left(r,T,\Theta_{L},\Theta_{s}\right)\}+A_{2}[\frac{1}{2}\left(1-\frac{\Theta_{s}}{\Theta_{L}}\right)+\frac{T}{\Theta_{L}}\ln\left(\frac{1-e^{-\frac{\Theta_{L}}{T}}}{1-e^{-\frac{\Theta_{s}}{T}}}\right)\\ & +\frac{\hbar c_{m}}{2rk_{B}\Theta_{L}}\left(\int_{0}^{\frac{rk_{B}\Theta_{s}}{\hbar c_{m}}}\frac{\sin x\;dx}{x}-\int_{0}^{\frac{rk_{B}\Theta_{L}}{\hbar c_{m}}}\frac{\sin x\;dx}{x}\right)-\frac{T}{\Theta_{L}}\Psi_{0}\left(r,T,\Theta_{L},\Theta_{s}\right)], \end{array}$$

where the two integrals:

(A2) $$\Psi_{1}\left(r,T,\Theta_{L},\Theta_{s}\right)=\int_{\frac{\Theta_{s}}{T}}^{\frac{\Theta_{L}}{T}}\frac{\sin\left(\frac{rk_{B}Tx}{\hbar c_{m}}\right)}{\left(\frac{rk_{B}Tx}{\hbar c_{m}}\right)}\frac{xdx}{e^{x}-1}$$

(A3) $$\Psi_{0}\left(r,T,\Theta_{L},\Theta_{s}\right)=\int_{\frac{\Theta_{s}}{T}}^{\frac{\Theta_{L}}{T}}\frac{\sin\left(\frac{rk_{B}Tx}{\hbar c_{m}}\right)}{\left(r\frac{k_{B}Tx}{\hbar c_{m}}\right)}\frac{dx}{e^{x}-1}$$

can be solved numerically or by series expansion of the argument, whereas:

(A4) $$\Theta_{s}=\frac{h\nu_{s}}{k_{B}}=\Theta_{D}{\left(\frac{4\pi }{3}\right)}^{\frac{1}{3}}\frac{1}{2\sqrt[{3}]{N}}$$

(A5) $$\Theta_{L}=\frac{h\nu_{L}}{k_{B}}=\Theta_{D}\left(1-\frac{\gamma b}{2\sqrt[{3}]{N}}\right)$$

(A6) $$A_{1}=\frac{6\hbar^{2}}{mk_{B}\Theta_{D}}{\left(1-\frac{\gamma b}{2\sqrt[{3}]{N}}\right)}^{2}$$

(A7) $$A_{2}=\frac{6\hbar^{2}\gamma b}{mk_{B}\Theta_{D}\sqrt[{3}]{N}}\left(1-\frac{\gamma b}{2\sqrt[{3}]{N}}\right),$$

with $b=\pi {\left(\frac{3}{4\pi }\right)}^{\frac{2}{3}}$ and $\gamma =\frac{1}{3}\left(1+\frac{2c_{l}^{2}}{c_{t}^{2}}\right){\left[3/\left(1+\frac{2c_{l}^{3}}{c_{t}^{3}}\right)\right]}^{\frac{2}{3}}$; *m* is the mass, *c_l_* and *c_t_* are, respectively, longitudinal and transversal sound speeds, from which the mean speed is obtained as $c_{m}=\sqrt[{3}]{\frac{3}{\left(c_{l}^{-3}+2c_{t}^{-3}\right)}}$.

Therefore, $\bar{\sigma_{}^{2}}\left(T,m,N,c_{l},c_{t},\Theta_{D}\right)$, that is, besides parameters characteristic of the specific metal (*m*, *c_l_* and *c_t_*) and temperature, the MSRD only depends on *N* and $\Theta_{D}$. For large crystals (i.e., for N→∞), $\Theta_{L}$ tends to $\Theta_{D}$, whereas $\Theta_{s}$ and $A_{2}$ tend to zero, and the expression for the traditional CD model [34] is obtained:

(A8) $$\begin{array}{ll} \bar{\sigma_{\infty }^{2}}= & \frac{6\hbar^{2}}{mk_{B}\Theta_{D}}\{\left[\frac{1}{4}+{\left(\frac{T}{\Theta_{D}}\right)}^{2}\int_{0}^{\frac{\Theta_{D}}{T}}\frac{xdx}{e^{x}-1}\right]+\frac{\cos\left(\frac{k_{B}\Theta_{D}r_{ij}}{\hbar c}\right)-1}{2{\left(\frac{k_{B}\Theta_{D}r_{ij}}{\hbar c}\right)}^{2}}\\ & -{\left(\frac{T}{\Theta_{D}}\right)}^{2}\int_{0}^{\frac{\Theta_{D}}{T}}\frac{\sin\left(\frac{k_{B}xr_{ij}T}{\hbar c}\right)}{\left(\frac{k_{B}r_{ij}T}{\hbar c}\right)}\frac{dx}{e^{x}-1}\}. \end{array}$$

In the present work, on Pd, m= 106.42 × 10^−3^ kg; *c_l_* = 4570 m/s and *c_t_* = 2060 m/s, respectively, for mean longitudinal and transversal sound velocity, with corresponding *c_m_* = 2323 m/s.

### Parametric Representation of the Static MSRD Component

The dependence of the static component of the MSRD, $\bar{\sigma_{0,r}^{2}}$, on the shell radius can be approximated by a simple one-parameter analytical expression. For truncated cubes the expression (Equation (7), reported again here for convenience) reads (see the schematic of Figure A1):

(A9) $$\bar{\sigma_{0,r}^{2}}=\{\begin{array}{cc} \sigma_{0,max}^{2}\left(r/D\right) & if\;0\leq r<D\\ \sigma_{0,max}^{2}\left(r-D_{max}\right)/\left(D-D_{max}\right) & if\;D\leq r<D_{max}\\ 0 & if\;r\geq D_{max} \end{array}$$

where *D* is the cube edge and *D_max_* is and maximum distance in the truncated cube, whereas $\sigma_{0,max}^{2}$ is the maximum of the static MSRD. Figure A2 shows examples for truncated cubes of different dimensions.

The use of the shape shown in Figure A1 is justified not only for a cube with 20% truncation, but in general for other values of truncation, as seen in Figure A3.

The quantity *D_max_* is trivial for standard shapes (i.e., it is the diameter for a sphere or √3D for a cube of edge D). Truncated cubes are geometrical shapes obtained by removing the edges and the vertices of a cube. The process of truncation can be formalized by using a truncation parameter p∈[0,1] so that 0% truncation (p=0) stands for perfect cube, while 100% truncation (p=1) is for the inscripted octahedron.

To determine the maximum distance inside a generic polyhedron, the common volume function (CVF) [42] is used. The CVF is the intersection volume between a body and the same body translated by a distance *L* along a direction [hkl] (see Figure A4)

Analytic expressions for the CVF of simpler shapes (like sphere or perfect cube) are available from the literature [43,44], and in general the CVF can be determined for any convex solids, also considering size dispersion (i.e., a distribution of sizes). In the context of MD simulations, the CVF is directly obtained numerically, by counting the atom couples at each distance L along the given [hkl] direction in the nanocrystal, so it is in principle known for any possible shape.

For any polyhedron of volume $V^{c}$, the $CVF_{hkl}$, is described by a cubic equation of *L* [27,42]. The value of *D_max_* (used in Equation (A9)) for any shape is; therefore, known from the literature on the CVF [27].
